# MRI in evaluation of perianal fistulae

**DOI:** 10.2478/v10019-010-0046-4

**Published:** 2010-10-14

**Authors:** Amela Sofic, Serif Beslic, Nedzad Sehovic, Jasmin Caluk, Damir Sofic

**Affiliations:** Institute of Radiology, Clinical Centre of University of Sarajevo, Sarajevo, Bosnia and Herzegovina

**Keywords:** perianal fistulae, X-rays, CT, MRI, fistulography, abscess

## Abstract

**Background:**

Fistula is considered to be any abnormal passage which connects two epithelial surfaces. Parks’ fistulae classification demonstrates the biggest practical significance and divides fistulae into: intersphincteric, transsphincteric, suprasphincteric and extrasphincteric. Etiology of perianal fistulae is most commonly linked with the inflammation of anal glands in Crohn’s disease, tuberculosis, pelvic infections, pelvic malignant tumours, and with the radiotherapy. Diagnostic method options are: RTG fistulography, CT fistulography and magnetic resonance imaging (MRI) of pelvic organs.

**Patients and methods:**

We have included 24 patients with perirectal fistulae in the prospective study. X-rays fistulography, CT fistulography, and then MRI of the pelvic cavity have been performed on all patients. Accuracy of each procedure in regards to the patients and the etiologic cause have been statistically determined.

**Results:**

29.16% of transphincteric fistulae have been found, followed by 25% of intersphincteric, 25% of recto-vaginal, 12.5% of extrasphincteric, and 8.33% of suprasphincteric. Abscess collections have been found in 16.6% patients. The most frequent etiologic cause of perianal fistulae was Crohn’s disease in 37.5%, where the accuracy of classification of MRI was 100%, CT was 11% and X-rays 0%. Ulcerous colitis was the second cause, with 20.9% where the accuracy of MRI was 100%, while CT was 80% and X-rays was 0%. All other etiologic causes of fistulae were found in 41.6% patients.

**Conclusions:**

MRI is a reliable diagnostic modality in the classification of perirectal fistulae and can be an excellent diagnostic guide for successful surgical interventions with the aim to reduce the number of recurrences. Its advantage is that fistulae and abscess are visible without the need to apply any contrast medium.

## Introduction

As per definition, a fistula is any abnormal passage connecting two epithelial surfaces. Anal fistulae have been known ever since the times of Hypocrates and have been described through centuries. In 1835, Frederick Salmon performed a successful operation in London on the writer Charles Dickens. Goodsall describes the fistulous passage in details, and Parks’ classification shows the most practical significance until nowadays. The classification refers to classifying fistulae on: intersphincteric, transsphincteric, suprasphincteric and extrasphincteric. In the more detail anatomical classification, the position of fistulae is used (“clockwise”) in respect to the clock hands to avoid any misinterpretation. Complex anal fistulae are those which are followed by risk factors: affected external sphincter anal muscle, forward location in women, multiple passages, incontinence, recurrences of fistulae, local radiation, chronic diarrhoea, Crohn’s disease.^1^

Aetiology of perianal fistulae is most commonly associated with the inflammation of anal glands in the Crohn’s disease, tuberculosis, pelvic infections, pelvic malignant tumours and with radiotherapy. Idiopathic fistulae are rare and are generally explained by chronic intramuscular anal infection (cryptoglandular hypothesis). In about 70% cases fistulous system drains through skin. Males are affected twice more than females, in ratio 2:1.^2^

### Scope of the study

The scope of the study is to indicate a possibility of pre-operative diagnostics with magnetic resonance imaging (MRI) in viewing and classifying perianal fistulae, with the aim to have as successful surgical treatment as possible, without recurrences. From the practical surgical point of view, it is of crucial importance to avoid incontinence as a post-operative complication.

## Patients and methods

From the 2008 to 2009 we included in the prospective study 24 patients with existing perirectal fistulae. The average age of patients was 41.45 ± 2.6 years, ranging from 19 to 67 years, with the same number of females 50% (n = 12) and males 50% (n = 12).

X-rays fistulography was performed first on all patients, followed by CT fistulography, and then by MRI of the pelvis. X-rays fistulography was performed on X-ray diascope (Practix 100, Philips, Aidhoven, the Netherlands), and after the application of Ultravist contrast medium through perianal fistulous openings. Immediately after that procedure, while the fistulous system was filled with the same contrast medium, the pelvic CT was performed on MDCT (Volume zoom, Siemens, Erlangen, Germany). The MRI pelvic examination was performed on 1.5 T machine (Avanto, Siemens, Erlagen, Germany) by using a routine protocol for pelvic examinations containing T1 3-plane views (axial, coronal and saggital planes), T2 axial and saggital planes, as well as bi-plane (axial and saggital planes) without the application of a contrast medium.

Based on the data obtained, a comparison of all findings has been done, using each particular method.

## Results

The following has been classified in 24 patients: transsphincteric fistulae in 29.16% of patients (n = 7), intersphincteric in 25% (n = 6), rectovaginal in 25% (n = 6), extrasphincteric in 12.5% (n=3) and suprasphincteric in 8.33% of patients (n = 2). Abscess collections were found in 16.6% of patients (n = 4) (Table 1).

Only 9 fistulae were identified by X-rays fistulography; 12 fistulae were identified by CT fistulography; and 20 by MRI (Table 1). For identifying fistulous ration in respect to the sphincter complex, MRI was a superior method, while for rectovaginal fistulae, CT and X-rays fistulography were better.

The most frequent cause of fistulae in our sample was Crohn’s disease with 37.5% (n = 9), followed by ulcerous collitis with 20.9% (n = 5), rectal cancer 16.7% (n = 4), postpartum 8.3% (n = 2), unknown aetiology 8.3% (n = 2), cervical cancer 4.2% (n = 1), and inflammatory dermoid cysts 4.2 % (n = 1) (Table 1).

X-rays fistulography demonstrated the accuracy of 37.5%. (χ^2^ = 4.444, p = 0.035), CT fistulography had accuracy of 50%, (χ2 = 6.000, p = 0.014), and the MRI demonstrated accuracy of 83.3%. (χ^2^ = 4.800, p = 0.028) (Table 2).

The comparison of results in respect to the sex of the patients demonstrated that there were differences in accuracy in favour of female patients (Table 2). Twenty-six % of all fistulae in our study are rectovaginal, where the total accuracy in females is on increase. The accuracy of X-rays fistulography in females was 75% vs. males 25%. The MRI accuracy for females was 66% vs. males 33%. Slightly higher accuracy for females was demonstrated in CT fistulography, in respect to the MRI, because it identified more rectovaginal fistulae compared to the MRI. The lowest accuracy was found in X-rays fistulography.

**FIGURE 1.**
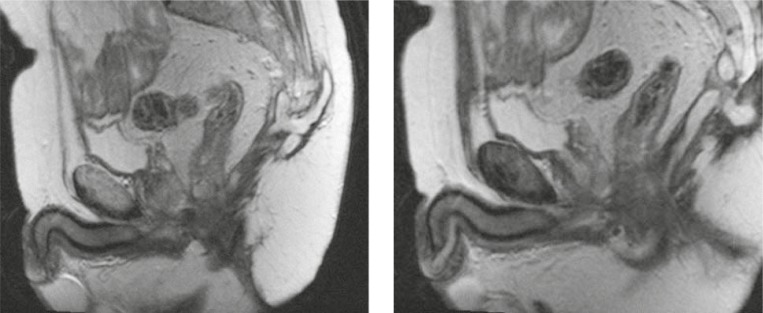
Suprasphincteric fistula with the abscess collection in the gluteal region (T2W, sagital).

**FIGURE 2.**
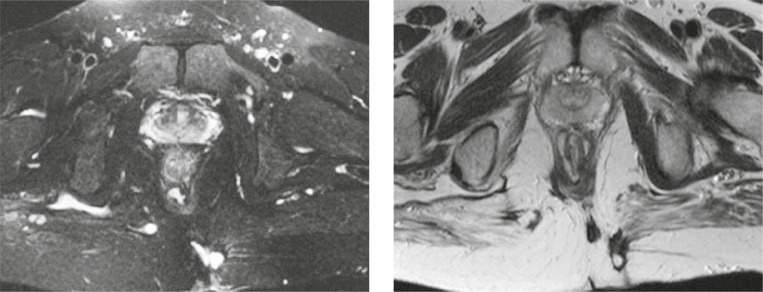
Intersphincteric fistula in the shape of a horseshoe with the opening to the left posterior (T2 Fs and T2W tra).

**FIGURE 3.**
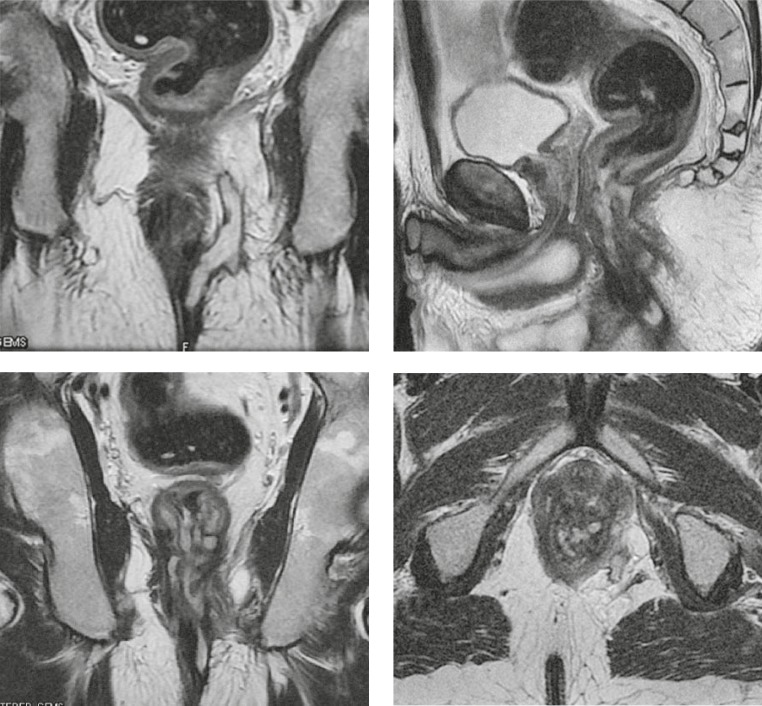
A complex transsphincteric fistula to the left (T2W cor,T2W sag,T2W tra).

The accuracy of procedures varies depending on the aetiology as well. X-rays fistulography demonstrates the accuracy in identifying: ulcerous colitis 60%, Crohn’s disease 0%, rectal cancer 75%, cervical cancer 0%, inflammatory dermoid cysts 100%, postpartum 100% and of unknown aetiology 0% (Table 3.). CT fistulography demonstrates the accuracy in identifying: ulcerous colitis 80%, Crohn’s disease 11.1%, rectal cancer 75%, cervical cancer 0%, inflammatory dermoid cyst 100%, postpartum 100%, and of unknown aetiology 50% (Table 4.). MRI demonstrates the accuracy in identifying: ulcerous colitis 100%, Crohn’s disease 100%, rectal cancer 50%, cervical cancer 100%, inflammatory dermoid cyst 100%, postpartum 0%, and of unknown aetiology 100% (Table 5.) (Figures 1, 2, 3).

## Discussion

Not so long time ago, surgeons performed operations on perirectal fistulae without the previous radiological assessment. The surgical examination under anaesthetics (EUA) consisted of visual inspection, palpation with the probe of a fistulous passage under general anaesthesia. Numerous diagnostic modalities failed in visualisation and classification of perianal fistulae.

Fistulography, as the earliest X-rays method, cannot classify fistulae due to the inadequate showing of anatomic structures, so that frequently it is unclear and difficult for the interpretation. CT can identify the existence of fistulous passages, either through non-ionic water solluble contrast media being inserted per rectum or through the fistulous opening. However, it is not sufficiend for a more detailed analysis of the whole complex of primary and numerous secondary branches in the fistulous system. Although the application of multidetector CT fistulography with the option of isotropic voxels and multiplaned imaging can bridge the aforementioned issues, researchers do not show enough interest in this field. It is obvious that the superiority of MRI and endorectal ultrasound (EUS) examination in the evaluation of perirectal fistulae provides a better motivation to the researchers.^3^

MRI with the superficial body-coil, besides other anatomic structures in the pelvis, shows excellent results in showing rectum, perianal region, internal, external anal sphincter, levator ani, ishiorectal and ishioanal region.^4^ In the evaluation of perirectal fistulae, it is very important to describe the relationship between fistula and the sphincteric mechanism in the coronal plane (Figure 3). It is equally important to describe the primary fistulous passage as well as the secondary ramification and possible associated abscess due to axial images. Images in the saggital plane are suitable for showing rectovaginal fistulae and the pre-sacral region. The surgical exploration without previous MRI diagnostics can be made difficult due to the presence of fibrosis and oedema, so that the MRI can identify the hidden intersphincteric space with the captured puss without rupturing the cutaneous layer. It is also useful in high fistulae – both transsphincteric and extrasphincteric. Fistulous passage, abscess, as well as all inflamed structures, show on T2W sequences high signal intensity, so they are easily noticed in respect to the muscles which have low signal intensity. On non-contrasted T1W sequences, fistulous passages (especially the secondary ones, the smaller ones), as well as abscess collections are identified with difficulties due to the moderate signal intensity of normal structures of the sphincter muscle and levatore ani. Therefore, it is recommended to use sequences with suppressed fats as the i.v. application of Gadolinium as a contrast medium. The fistulous opening on skin can be filled with Gadolinium, thus obtaining the picture of fistulous system in hipersignal in T1W time, or by a cheaper method – by injecting physiological solution with showing the fistulous system in hipersignal in T2W time.

There is not a large number of published studies in relation to the fistulography. Thus, Kuipers and Schulpen as far as in 1985 emphasized that fistulography is a reliable and accurate method in only 16% cases.^5^ The best results were obtained by Weisman *et al.* who managed to obtain reliable fistulography in half of the examined cases – 27 patients in his study, which generally represents only very modest results.^6^

The first reporting on the accuracy of MRI in detection and classification of perianal fistulae was from 1992 – 1994 in Lunnis’ publications, with the matching of 86–88% between MRI and surgical findings.^7^,^8^ EUS is not a comfortable procedure, however, it has a very good spatial resolution due to the close contact of the probe (10MHZ) with the rectal wall, which gives it the superiority in evaluation of fistulous openings within the rectum and the level of disruption of the sphincter in post-surgically incontinent patients. MRI is more comfortable^4^ and better shows intersphincteric abscesses. Halligan and Stoker claim that MRI assists the surgeon to reduce post-surgical recurrences for 75%, and that the EUS is only an alternative when it is not possible to perform the MRI.^9^ Hussain *et al.* from the Netherlands, in his study from 1996, talked about the superiority of MRI in classification of fistulae compared to the EUS (89 vs 61%).^10^ Spenser’s study from 1996, in which body-coil was used, demonstrated the accuracy of the MRI in showing perirectal fistulae of 88%.^11^ The same author, two years later, in 1988, obtained the classification of fistulae by EUS of 81%, and by the MRI of 90%.^12^ Sensitivity of the primary fistulous passage reaches up to 100%, however, with the specificity of 86%. Yee *et al.* concludes in his study that the native endoscopic ultrasound does not detect rectovaginal fistulae.^13^

In the recent times, ultrasound visibility of fistulae filled with peroxide has been researched, which is strictly recommended for the pre-surgical evaluation in patients with recurrences of fistulae. Gustafson *et al.* claimed that the endoscopic EUS complemented the MRI in classification of fistulae.^14^

The optimal diagnostic approach is a combination of EUS, examination under anaesthesia (EUA) and MRI in patients with fistulae who have the Crohn’s disease, reports Schwartz *et al.* in his article.^15^ The Viennese group of radiologists, in their study, obtains the accuracy in classification of fistulae by the MRI of 84%, vs EUS of 60%.^16^

Gravante and Giordano in 2008 promoted 3D EUS as equal to the MRI in the examination of fistulae. The accuracy of the MRI was obtained in the range of 90 % compared with the EUS which obtained 81%.^17^ Schratter-Sehn *et al.* obtained higher sensitivity by the EUS of 82% compared to n CT of 24% in the classification of perianal fistulae in the Crohn’s disease, while Schaefer *et al.* obtains the matching with the surgical findings of 89%.^18^,^19^ In our study, we obtained the accuracy in classification of fistulae by X-rays of 37.5%, with the CT accuracy was 50%, and the MRI accuracy was 83%. The most frequent aetiological cause of perianal fistulae was Crohn’s disease of 37.5%, where the accuracy of classification by the MRI was 100%, by CT of 11% and by X-rays was 0%. Ulcerous colitis was on the second place with 20.9%, where the accuracy in that respect was 100% by the MRI, 80% by CT and 0% by X-rays. All other etiological causes of fistulae including radiation, which is a serious side effect after oncological treatment^20^,^21^, were 41.6%. Abscess collection was found in 16.6 patients.

## Conclusions

MRI is a reliable diagnostic modality in the classification of perirectal fistulae and can be an excellent diagnostic guide for successful surgical interventions with the aim to reduce the number of recurrences. Its advantage is that fistulae and abscess are visible without the need to apply any contrast medium.

## Figures and Tables

**TABLE 1.** Fistulae classification and the etiology

**Patient**	**age**	**sex**	**etiology**	**Type of fistulae**	**RTG**	**CT**	**MRI**	**Absces**
**1**	34	M	Crohn’s disease	**Intersphincteric**			+	
**2**	25	M	Crohn’s disease	**Transsphincteric**			+	
**3**	38	M	Crohn’s disease	**Transsphincteric**			+	
**4**	56	F	Ca cerv-radiation	**Transsphincteric**			+	
**5**	33	M	Crohn’s disease	**Transsphincteric**			+	
**6**	67	F	Crohn’s disease	**Intersphincteric**			+	
**7**	28	M	Crohn’s disease	**Transsphincteric**			+	
**8**	33	M	Crohn’s disease	**Transsphincteric**			+	
**9**	25	F	Unknown	**Intersphincteric**			+	
**10**	44	M	Unknown	**Suprassphincteric**		**+**	+	+
**11**	56	F	Crohn’s disease	**Suprassphincteric**		**+**	+	+
**12**	56	F	Postpartum	**Rectovaginal**	+	**+**		
**13**	45	F	Ca recti	**Rectovaginal**	+	**+**		
**14**	31	M	Ulcerous colitis	**Intersphincteric**			+	
**15**	19	M	Ulcerous colitis	**Extrasphincteric**	+	**+**	+	
**16**	55	F	Ca recti-radiation	**Rectovaginal**	+	**+**	+	
**17**	45	F	Infla.dermoid cysts	**Rectovaginal**	+	**+**	+	+
**18**	30	F	Postpartum	**Rectovaginal**	+	**+**		
**19**	34	F	Ulcerous colitis	**Extrasphincteric**	+	**+**	+	+
**20**	44	F	Ulcercous colitis	**Transsphincteric**		**+**	+	
**21**	42	M	Ulcerous colitis	**Extrasphincteric**	+	**+**	+	
**22**	54	M	Ca recti	**Intersphincteric**			+	
**23**	41	M	Crohn’s disease	**Intersphincteric**			+	
**24**	60	F	Ca recti	**Rectovaginal**	+	**+**		

**Accuracy**					**9**	**12**	20	4
					37,5%	50%	83%	16,6%

**TABLE 2.** Accuracy of all three procedures

	**X-rays**					**CT**			**MRI**		

			**Sex**		**Total**	**Sex**		**Total**	**Sex**		**Total**
			**M**	**F**		**M**	**F**		**M**	**F**	
						
**X-rays**	**Neg. (−)**	**Number**	10	5	15	9	3	12	0	4	4
		**%**	83.3	41.7	62.5	75.0	25.0	50.0	0.0	33.3	16.7
	**Pos. (+)**	**Number**	2	7	9	3	9	12	12	8	20
		**%**	16.7	58.3	37.5	25,0	75,0	50.0	100,0	66,7	83.3
	
**Total**		**Number**	12	12	24	12	12	24	12	12	24
		%	50.0	50.0	100.0	50.0	50.0	100.0	50.0	50.0	100.0

			χ^2^=4.444	p=0.035		χ^2^=6.000	p=0.014		χ^2^=4.800	p=0.028	

**TABLE 3.** The accuracy of procedures varies depending on the aetiology – X-rays

**X-rays**										

			**Aetiology**							**Total**

			**Cervical cancer**	**Rectal cancer**	**Inflammatory dermoid cysts**	**Crohn’s disease**	**Unknown**	**Postpartum**	**Ulcerous colitis**	
	**Neg. (−)**	**Number**	1	1	0	9	2	0	2	15
		**%**	100.0	25.0	0.0	100.0	100.0	0.0	40.0	62.5
	**Pos. (+)**	**Number**	0	3	1	0	0	2	3	9
		**%**	0.0	75.0	100.0	0.0	0.0	100.0	60.0	37.5

**Total**		**Number**	1	4	1	9	2	2	5	24
		**%**	100.0	100.0	100.0	100.0	100.0	100.0	100.0	100.0
		
			χ^2^=17.600	p=0.014						

**TABLE 4.** The accuracy of procedures varies depending on the aetiology – CT

**CT**										

			**Aetiology**							**Total**

			**Cervical cancer**	**Rectal cancer**	**Inflammatory dermoid cysts**	**Crohn’s disease**	**Unknown**	**Postpartum**	**Ulcerous colitis**	
	**Neg. (−)**	**Number**	1	1	0	8	1	0	1	12
		**%**	100.0	25.0	0.0	88.9	50.0	0.0	20.0	50.0
	**Pos. (+)**	**Number**	0	3	1	1	1	2	4	12
		**%**	0.0	75.0	100.0	11.1	50.0	100.0	80.0	50.0

**Total**		**Number**	1	4	1	9	2	2	5	24

		**%**	100.0	100.0	100.0	100.0	100.0	100.0	100.0	100.0

			χ^2^=14.444	p=0.087						

**TABLE 5.** The accuracy of procedures varies depending on the aetiology – MRI

**MR**										

			**Aetiology**							**Total**

			**Cervical cancer**	**Rectal cancer**	**Inflammatory dermoid cysts**	**Crohn’s disease**	**Unknown**	**Postpartum**	**Ulcerous colitis**	
	**Neg. (−)**	**Number**	0	2	0	0	0	2	0	4
		**%**	0.0	50.0	0.0	0.0	0.0	100.0	0.0	16.7
	**Pos. (+)**	**Number**	1	2	1	9	2	0	5	20
		**%**	100.0	50.0	100.0	100.0	100.0	0.0	100.0	83.3

**Total**		**Number**	1	4	1	9	2	2	5	24
		**%**	100	100.0	100.0	100.0	100.0	100.0	100.0	100.0

			χ^2^=16.800	p=0.019						
